# Simulations of lipid bilayers using the CHARMM36 force field with the TIP3P-FB and TIP4P-FB water models

**DOI:** 10.7717/peerj.5472

**Published:** 2018-08-14

**Authors:** Fatima Sajadi, Christopher N. Rowley

**Affiliations:** Department of Chemistry, Memorial University of Newfoundland, St. John’s, NL, Canada

**Keywords:** Lipid, Molecular dynamics, Force field, Water model, Molecular simulation, Head group area, Scattering, Membrane permeability

## Abstract

The CHARMM36 force field for lipids is widely used in simulations of lipid bilayers. The CHARMM family of force fields were developed for use with the mTIP3P water model. This water model has an anomalously high dielectric constant and low viscosity, which limits its accuracy in the calculation of quantities like permeability coefficients. The TIP3P-FB and TIP4P-FB water models are more accurate in terms of the dielectric constant and transport properties, which could allow more accurate simulations of systems containing water and lipids. To test whether the CHARMM36 lipid force field is compatible with the TIP3P-FB and TIP4P-FB water models, we have performed simulations of 1,2-dipalmitoyl-sn-glycero-3-phosphocholine and 1-palmitoyl-2-oleoyl-sn-glycero-3-phosphocholine bilayers. The calculated headgroup area, compressibility, order parameters, and X-ray form factors are in good agreement with the experimental values, indicating that these improved water models can be used with the CHARMM36 lipid force field without modification when calculating membrane physical properties. The water permeability predicted by these models is significantly different; the mTIP3P-model diffusion in solution and at the lipid–water interface is anomalously fast due to the spuriously low viscosity of mTIP3P-model water, but the potential of mean force of permeation is higher for the TIP3P-FB and TIP4P-FB models due to their high excess chemical potentials. As a result, the rates of water permeation calculated the FB water models are slower than the experimental value by a factor of 15–17, while simulations with the mTIP3P model only underestimate the water permeability by a factor of 3.

## Introduction

Realistic molecular dynamics (MD) simulations of lipid-containing systems like bilayers, vesicles, and membrane–protein systems require accurate molecular mechanical force fields for lipids. These models have been carefully parameterized using ab initio data and the empirical properties of bilayers. A variety of lipid models have been developed, including the Berger (Berger, Edholm & Jähnig, 1997), Slipids (Jämbeck & Lyubartsev, 2012), and the CHARMM models (Klauda et al., 2010; Pastor & MacKerell, 2011). The performance of these models is evaluated based on their ability to predict empirical data regarding the structure and dynamics of lipid properties.

A common practice in evaluating force fields has been to evaluate the ability of these models to predict physical descriptors of lipid bilayers (Venable, Brown & Pastor, 2015). The headgroup area (*A*_L_) corresponds to the average surface occupied by one lipid in the bilayer. The area compressibility (*K*_A_) of the bilayer indicates the energetic cost of an elastic expansion of the bilayer surface area. Poger, Caron & Mark (2016) noted that these properties are inferred from several different experimental techniques, so the range of reported experimental values can be broad, including for lipids that are commonly used in simulations.

Scattering experiments have provided more direct data to validate the ability of force fields to predict the structure of bilayers (Lewis & Engelman, 1983; Nagle & Tristram-Nagle, 2000; Kučerka, Katsaras & Nagle, 2010; Kučerka et al., 2008; Kučerka, Tristram-Nagle & Nagle, 2006b, Kučerka, Nieh & Katsaras, 2011). Form factors from X-ray scattering experiments can be used to infer the transmembrane electron density distribution (ρ(*z*)), which is particularly sensitive to the position of phosphates of the lipid headgroups. Neutron scattering can be used to calculate neutron scattering length density (NSLD) profiles as a function of bilayer depth. The NSLD profiles from neutron scattering experiments are performed with D_2_O because there is a sharp difference between the scattering lengths of the aqueous deuterons and the lipid protons, providing a measure of the hydrophobic thickness of the bilayer. The X-ray and neutron scattering profiles calculated from MD simulations can be compared to the profiles inferred from these experiments or the experimental and calculated form factors can be compared directly (Kučerka, Katsaras & Nagle, 2010).

Order parameters are another method for validating lipid models (Vermeer et al., 2007). The order parameters (*S*_CH_) of the headgroups and acyl chains of the lipid tails provide a measure of the configurational flexibility of the lipids tails as a function of position along the lipid chain. These parameters can be determined from the nuclear magnetic resonance (NMR) coupling constants of lipids, providing an experimental test of the predicted conformational flexibility of the lipids from the water–lipid interface to the center of the bilayer.

The development of lipid models is ongoing because some properties of lipid bilayers have proven difficult to predict accurately using existing models. For example, established non-polarizable force fields typically overestimate the membrane dipole potential (MDP, ϕ(*z*)) of lipid bilayers (Harder, MacKerell & Roux, 2009). This potential arises from changes in the electrostatic potential between the solution and the various components of the bilayer and reflects the average strength of interaction of a test charge at different bilayer depths (Wang, 2012).

The CHARMM36 lipid model has proven to be quantitatively accurate for lipid bilayer properties such as thickness, headgroup area, order parameters, and form factors (Klauda et al., 2010; Pastor & MacKerell, 2011). Simulations of lipids require the selection of a model for the water molecules in the system. The CHARMM36 lipid model was parameterized for use with the mTIP3P water model. This water model underestimates the viscosity of liquid water (González & Abascal, 2010), resulting in spuriously high rates of self-diffusion. This model also overestimates the dielectric constant of water (Braun, Boresch & Steinhauser, 2014), so the physical description of the partitioning of charged or polar solutes between the aqueous solution and the bilayer interior is imperfect. These issues are sources of error in quantitative calculations of some membrane processes, particularly for transport properties like the rate of permeation of water and other solutes across a bilayer (Gumbart et al., 2013; Lee et al., 2016a; Orsi & Essex, 2010).

The predicted properties of liquid water varies significantly with how the molecular mechanical model represents the structure and intermolecular interactions of water molecules (Brini et al., 2017; Onufriev & Izadi, 2017). The TIP3P model was developed in 1983 by Jorgensen et al. (1983). The model features partial atomic charges centered on the hydrogen and oxygen atoms and holds a rigid geometry that is consistent with water in the liquid phase (∠HOH = 104.5°, *r*_OH_ = 0.9572 Å). In the original TIP3P model, there is a single Lennard-Jones interaction potential between oxygen atoms, but there is a popular variant where there are also Lennard-Jones sites on the hydrogen atoms. This model is referred to as the mTIP3P, TIPS3P, or CHARMM TIP3P model. The mTIP3P model has been used in the development of the CHARMM force fields, so this model has been prescribed for use in simulations using these force fields. The mTIP3P model is used in the simulations reported in this paper.

The TIP3P/mTIP3P models predate computational algorithms such as Particle Mesh Ewald (PME) electrostatics, so these parameters are not optimal for use with modern simulation methods. Although this simple model performs reasonably well for predicting the density and enthalpy of vaporization of water under ambient conditions, its dielectric constant and diffusivity coefficients are anomalously high (Table 1). Many alternative water models have been developed that describe the physical properties of water more realistically. In the TIP4P model, a fourth charge site is added along the HOH bisector. The original TIP4P model has been reparameterized several times, including the development of the TIP4P-Ew and TIP4P/2005 models (Vega, Abascal & Nezbeda, 2006). These models describe many of the physical properties of water with remarkable accuracy, although the dielectric constants were systematically lower than the experimental values (ε ≈ 60). More complex models with additional charge sites have been defined (e.g., TIP5P), although they have not become widely adopted in MD simulations. Despite the existence of models that describe the properties of water more accurately, TIP3P and mTIP3P have remained the mainstay in biomolecular simulation. This is because established force fields like CHARMM36 have not been comprehensively validated for use with other water models and bilayer properties can be sensitive to the water model used. Piggot, Piñeiro & Khalid (2012) found that 1,2-dipalmitoyl-sn-glycero-3-phosphocholine (DPPC) lipid bilayers underwent a phase transition to a tilted gel phase when simulated with the CHARMM36 force field, the TIP3P water model, and energy-based non-bonded switching functions instead of the CHARMM-type force-based non-bonded switching functions with mTIP3P water. Recently, Javanainen et al. (2018) showed that the CHARMM36 lipid force field predicted the properties of 1-palmitoyl-2-oleoyl-sn-glycero-3-phosphocholine (POPC) and DPPC monolayers accurately when used with the four-point OPC model, (Izadi, Anandakrishnan & Onufriev, 2014) suggesting that it may be possible to use the existing CHARMM36 lipid model with more accurate water models.

**Table 1:** Physical properties of water predicted by the mTIP3P, TIP3P-FB, and TIP4P-FB water models (298.15 K, 101.325 kPa).

Property	Expt.	mTIP3P	TIP3P-FB	TIP4P-FB
Density (ρ)/g cm^−3^	0.997	0.98	0.995	0.996
Enthalpy of vaporization (Δ*H*_vap_)/kcal mol^−1^	10.52	9.81	10.71	10.80
Dielectric constant (ε)	78.5	104	81.3	77.3
Diffusivity (*D*)/10^−5^ cm^2^ s^−1^	2.29	6.48	2.28	2.21

**Note:**

TIP3P-FB and TIP4P-FB values are reproduced from Wang, Martinez & Pande (2014).

**Figure 1:**
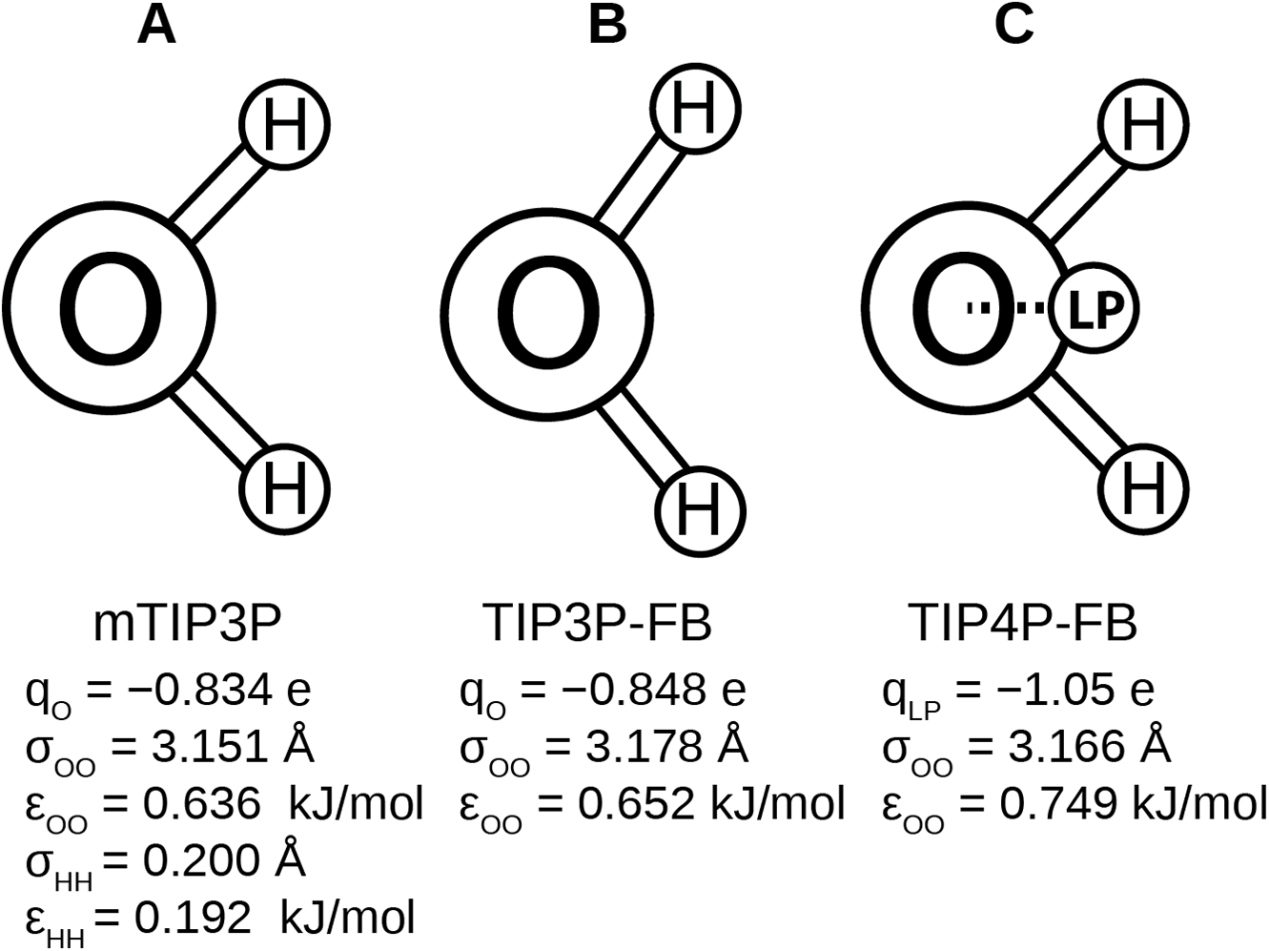
Schematics of the (A) mTIP3P, (B) TIP3P-FB, and (C) TIP4P-FB water models. The electrostatic and Lennard-Jones parameters are listed beneath each model.

Recently, Wang, Martinez & Pande (2014) developed the ForceBalance code, which allows the parameters of a molecular mechanical model to be optimized systematically. This code was used to develop the TIP3P-FB and TIP4P-FB water models, which were parameterized to reproduce the enthalpy of vaporization, density, dielectric constant, isothermal compressibility, heat capacity, and thermal expansion coefficient of liquid water. Like the mTIP3P model, the TIP3P-FB model has partial atomic charges on each of the nuclear centers, but the ∠HOH = 103.5° angle is increased to 108.1° and the O–H bond length is increased to 1.01 Å. The TIP4P-FB model holds the same molecular geometry as TIP3P, but the charge on the oxygen atom is shifted to a virtual site located on the bisector of the ∠HOH. (Fig. 1). For all three models, there is a Lennard-Jones interaction site that is centered at the oxygen atom,

(1) $${\cal V}(r) = 4{{\rm{\varepsilon }}_{{\rm{OO}}}}\left[ {{{\left({{{{{\rm{\sigma }}_{{\rm{OO}}}}} \over r}} \right)}^{12}}-{{\left({{{{{\rm{\sigma }}_{{\rm{OO}}}}} \over r}} \right)}^6}} \right]$$

where ɛ_OO_ and σ_OO_ are the well-depth and atomic radius parameters. The mTIP3P model also has weakly-interacting Lennard-Jones potentials for atomic pairs involving the hydrogen atoms.

TIP3P-FB and TIP4P-FB water models accurately describe many of the physical properties of water, including its viscosity and dielectric constant. Further, the TIP4P-FB model predicts the variation of these properties with temperature more accurately but does not underestimate the dielectric constant like other four-point water models (ɛ = 78). Using the same procedure, the ForceBalance method can be used to develop force field parameters for other components, providing a systematic route to develop improved force fields that are based on the FB water models. Lipid simulations using improved water models, like TIP3P-FB or TIP4P-FB, could provide more accurate descriptions of phenomena like transport properties and partitioning, but it has not been shown that these models are compatible. Lipid bilayer properties are sensitive to effects like headgroup solvation, so changing the water model could cause the bilayer properties predicted by these simulations to be less accurate.

**Figure 2:**
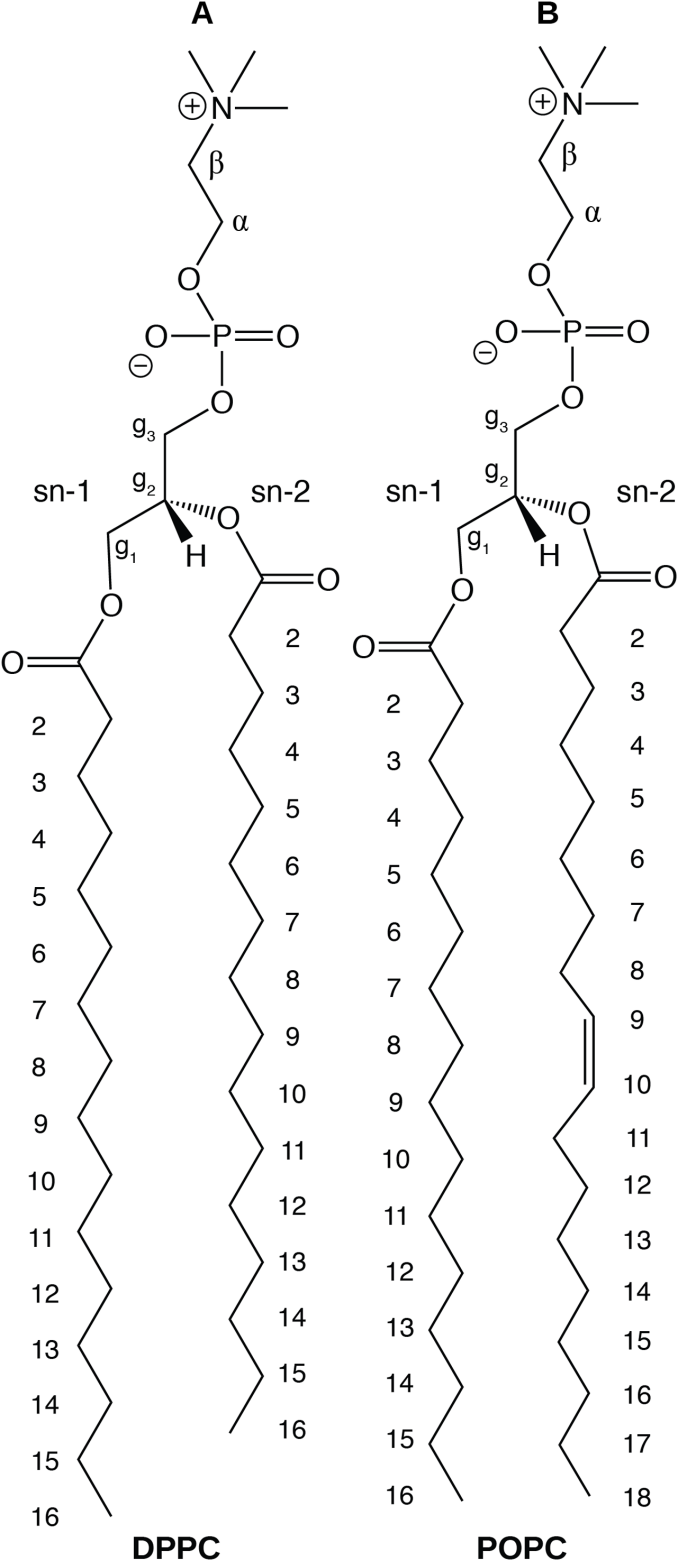
Chemical structure of (A) DPPC and (B) POPC, the two lipids modeled in this study.

In this paper, we report physical and structural properties of lipid bilayers described using the CHARMM36 lipid model, in combination with the mTIP3P, TIP3P-FB, and TIP4P-FB water models. The lipid headgroup areas, electron density profiles, X-ray form factors, MDP, and order parameters were calculated from these simulations and used to model the lipid bilayers. DPPC, a saturated lipid, and POPC, an unsaturated lipid, were used to test these models (Fig. 2). These lipids were chosen because they are commonly used in simulation and experimental studies of model bilayers and there is extensive experimental data on their properties. We also calculate the potential of mean force (PMF) and diffusivity profiles of water molecules permeating across a POPC bilayer using these three water models to calculate the membrane permeability.

## Computational Methods

### Lipid bilayer simulations

**Figure 3:**
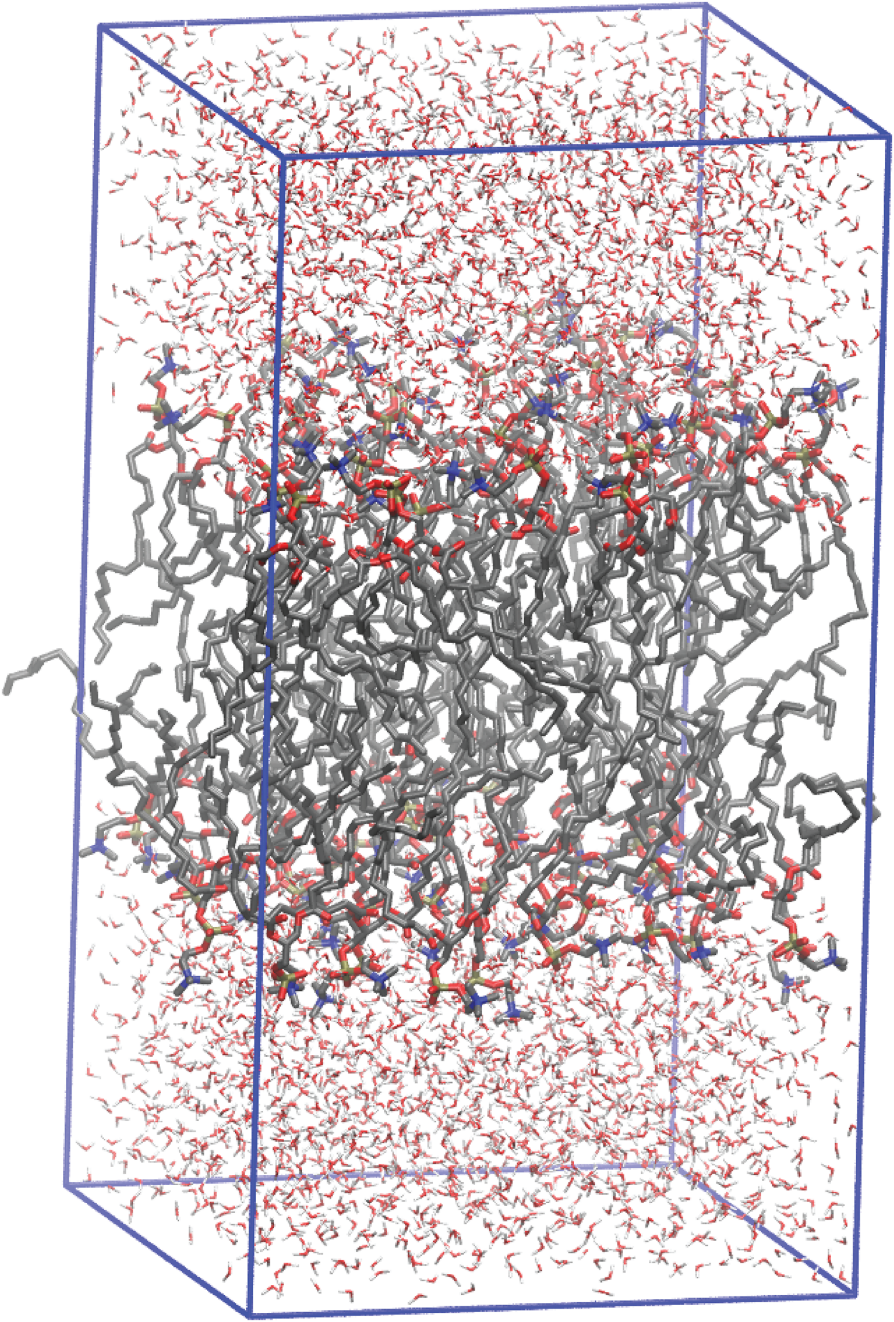
A rendering of the simulation cell used in the simulations of POPC.

Pure DPPC and POPC bilayers were constructed using the Membrane Builder feature of the CHARMM-GUI interface (Jo, Kim & Im, 2007; Jo et al., 2008, 2009; Wu et al., 2014; Lee et al., 2016b). All simulations were performed using NAMD 2.12 (Phillips et al., 2005). A 2 fs time step was used. Properties were calculated from the average of three simulations that were each 500 ns in length, following 100 ns equilibration simulations. Uncertainties of the calculated properties were calculated using the largest uncertainty of the three simulations calculated using block averaging (Venable, Brown & Pastor, 2015; Allen & Tildesley, 1989). The DPPC simulation cell contained 74 lipids and 4,241 water molecules, while the POPC simulation cell contained 68 lipids and 4,253 water molecules. The approximate dimensions of the simulation cells were 44 × 44 × 110 Å. An example simulation cell is depicted in Fig. 3. DPPC bilayers were simulated at 323 K, while the POPC bilayers were simulated at 303 K.

Lennard-Jones interactions were scaled to zero at a distance of 12 Å. CHARMM-style force-based switching was applied to the Lennard-Jones potential (i.e., the “vdwForceSwitching on” option in NAMD). The CHARMM36 lipid models were developed using this switching function and simulations using different treatments of non-bonded interactions can result in significant differences in lipid bilayer properties (Piggot, Piñeiro & Khalid, 2012). Long-range electrostatic interactions were described using the PME method (Darden, York & Pedersen, 1993) with a grid spacing of one Å. Bonds containing hydrogen atoms were kept rigid using the SHAKE algorithm (Ryckaert, Ciccotti & Berendsen, 1977). The bilayers were simulated under isothermal-isobaric conditions (i.e., no applied surface tension) using a Langevin thermostat with a friction coefficient of 1 ps^−1^ and a Nosé–Hoover Langevin piston barostat with a decay period of 50 fs. Sample input files are provided in the Supplementary Information. The lipids were represented using the CHARMM36 parameters for DPPC and POPC, while the water molecules were represented using the mTIP3P (Jorgensen et al., 1983; Durell, Brooks & Ben-Naim, 1994; Neria, Fischer & Karplus, 1996), TIP3P-FB, and TIP4P-FB models (Wang, Martinez & Pande, 2014). Electron density and neutron scattering profiles were calculated using the Density Profile extension (Giorgino, 2014) of Visual Molecular Dynamics (VMD) and transformed into reciprocal space using the theory described by Benz et al. (2005) and atomic parameters of the SimToExp code (Kučerka, Katsaras & Nagle, 2010).

The average headgroup area was calculated from the average of the X–Y area (calculated from the *X* and *Y* lengths of the cubic simulation cell: *L_X_* and *L_Y_*) and the number of lipids per leaflet (*n_L_*),

(2) $${A_L} = {{\langle {L_X}{L_Y}\rangle } \over {{n_L}}}$$

The compressibility was calculated from the fluctuation of the headgroup area in an equilibrium simulation,

(3) $${K_A} = {{{k_B}T\langle {A_L}\rangle } \over {{n_L}\langle {\rm{\delta }}A_L^2\rangle }}$$

### Order parameters

The order parameters were calculated using the MEMPLUGIN (Guixà-González et al., 2014) extension of VMD 1.9.2 (Humphrey, Dalke & Schulten, 1996) and the calcOrderParameters script of the NMRlipids project (Botan et al., 2015). The order parameters are calculated from the angle (θ) formed between the designated C–H bond and a vector normal to the surface of the bilayer using the relation,

(4) $${S_{{\rm{CH}}}} = \left| {\left\langle {{{3\mathop {\cos }\nolimits^2 ({\rm{\theta }})-1} \over 2}} \right\rangle } \right|$$

### Membrane dipole potential

The MDP was calculated from the charge density along the transmembrane axis of the simulation cell (ζ(*z*)) averaged over the length of the simulations. The MDP was calculated numerically from these data using the relation,

(5) $$\phi (z)-\phi ({z_0}) =-{1 \over {{{\rm{\varepsilon }}_0}}}\int_{{z_0}}^z \int_{{z_0}}^{z'}\hskip-3.5pt{\rm{\zeta }}(z'')dz''dz'.$$

*z*_0_ is a position in solution where ϕ(*z*) should be zero. The charge density was calculated from an average of the partial atomic charges centered at the nuclei.

### Permeability

Several theoretical models have been developed to predict the rates of permeation from molecular simulations, although the solubility-diffusion model has been particularly popular (Marrink & Berendsen, 1994, 1996; Riahi & Rowley, 2014; Lee et al., 2016a; Awoonor-Williams & Rowley, 2016). This model predicts the permeability coefficient (*P_m_*) from the PMF (*w*(*z*)) and the diffusivity (*D*(*z*)) of the permeating solute as a function of its position, *z*, along the transmembrane axis,

(6) $${1 \over {{P_m}}} = \int_{-L/2}^{L/2} {{{{\rm{e}}^{w(z)/{k_B}T}}} \over {D(z)}}{\rm{d}}z$$

The PMF was calculated using an umbrella sampling simulation, where the position of one water molecule was restrained to a designated position with respect to the center of mass of the bilayer along the *z*-axis. A harmonic restraint with a spring constant of 2.5 kcal/mol Å^−2^ was used to restrain the solute. The initial windows were generated by placing the permeating water molecule at the designated position along the *Z*-axis and a random position on the X–Y plane. The bilayer structure for each window was selected from a random frame from the long-timescale bilayer simulations. A 120 ns MD simulation was performed for each window, where the first 20 ns was discarded as equilibration. Although the calculation of the PMF of permeation for charged solutes like arginine requires extremely long simulations (e.g., μs) (Neale et al., 2011), the PMF for permeation of small neutral solutes can be calculated from these comparatively short simulations (Nitschke, Atkovska & Hub, 2016). The PMF was calculated from these windows using the Weighted Histogram Analysis Method (WHAM) (Roux, 1995; Kumar et al., 1992; Grossfield, 2018). The diffusivity profile was calculated from the average of three 2 ns NVE simulations where the solute was restrained using a 20 kcal/mol Å^−2^ harmonic force constant. The diffusivity was calculated from these time series using generalized Langevin analysis of the position autocorrelation function of these time series (Hummer, 2005; Gaalswyk, Awoonor-Williams & Rowley, 2016).

### Calculation of transfer energies

The transfer energies and excess chemical potentials were calculated using the staged thermodynamic-integration/free-energy-perturbation technique of Deng & Roux (2004). The electrostatic component was calculated by scaling the solute charges to zero through scaling factors of λ = [0.0, 0.1, 0.2, 0.3, 0.4, 0.5, 0.6, 0.7, 0.8, 0.9, 1.0]. The dispersion and repulsive components were calculated using a Weeks–Chandler–Andersen decomposition of the Lennard-Jones terms of the solute. The dispersion component was calculated through an 11-window thermodynamic integration calculation with λ = [0.0, 0.1, 0.2, 0.3, 0.4, 0.5, 0.6, 0.7, 0.8, 0.9, 1.0]. The repulsive component was calculated using a nine-stage free energy perturbation calculation. Each window/stage of the simulation was simulated for 1 ns for equilibration following by 2 ns of sampling. Replica exchange was used to allow exchanges between neighboring windows of the TI/FEP simulation at two ps intervals, following the procedure of Jiang et al. (2014). Gibbs energies were calculated using WHAM (Kumar et al., 1992).

## Results

### Headgroup area and compressibility

The headgroup areas and compressibilities of the bilayer models calculated from the MD simulations are presented in Tables 2 and 3, respectively. Although all three water models give headgroup areas within a 1.2 Å range, a Student’s *t*-test shows the distributions are statistically distinct (*p* < 0.0001) for all pairs of distributions. The TIP3P-FB water model tends to predict smaller headgroup areas, which puts it into closer agreement with experiment than the mTIP3P model for the DPPC bilayer but worse agreement for the POPC bilayer. The mTIP3P and TIP4P-FB models give similar headgroup areas. The DPPC compressibility predicted by the FB models are larger and in better agreement with the experimental value. The POPC compressibilities are less systematic, as the TIP4P-FB model predicts a lower compressibility than the mTIP3P model but the TIP3P-FB model predicts a higher compressibility.

**Table 2:** Lipid headgroup areas for DPPC and POPC bilayers in Å^2^.

Water model	DPPC	POPC
mTIP3P	61.1 ± 0.9	64.6 ± 1.0
TIP3P-FB	60.3 ± 0.9	64.0 ± 0.9
TIP4P-FB	61.5 ± 1.2	65.2 ± 1.1
Expt.	63.1 ± 1.3	64.3 ± 1.3

**Note:**

Experimental values are taken from Kučerka, Nieh & Katsaras (2011). The uncertainties of the calculated values are calculated from the standard deviation of the results from three independent simulations of the bilayer.

**Table 3:** Compressibility for DPPC and POPC bilayers in dyne/cm.

Water model	DPPC	POPC
mTIP3P	189.8 ± 8.00	237.2 ± 10.60
TIP3P-FB	265.8 ± 38.39	264.8 ± 9.99
TIP4P-FB	230.5 ± 10.40	214.6 ± 10.23
Expt.	231^a^	180–330^b^

**Notes:**

The uncertainties of the calculated values are calculated from the standard deviation of the results from three independent simulations of the bilayer.

a Nagle & Tristram-Nagle (2000).

b Binder & Gawrisch (2001).

### C–H order parameters

**Figure 4:**
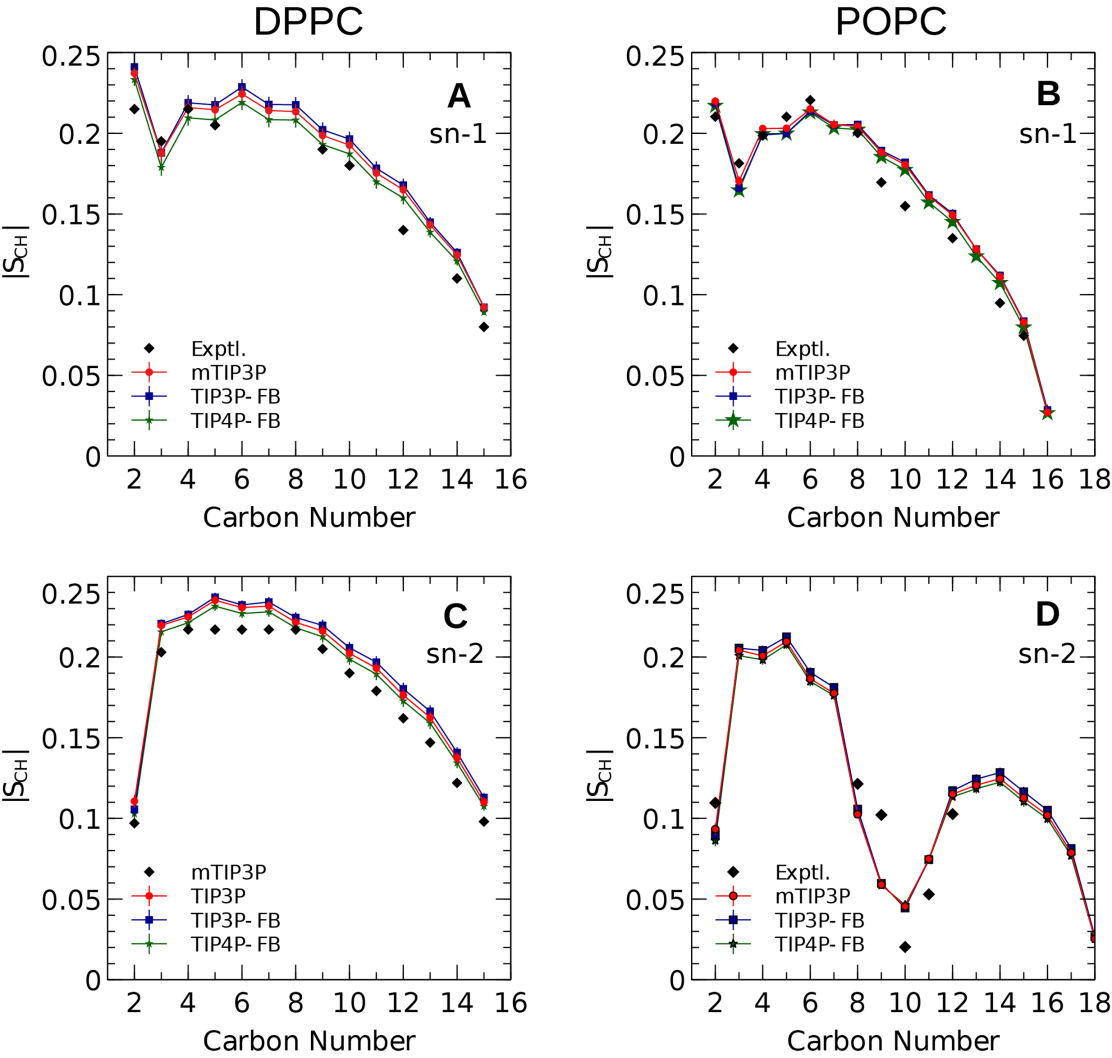
NMR deuterium order parameters (|*S_CD_*|) for the lipid tails of the DPPC and POPC bilayers calculated from simulations of the bilayers with the mTIP3P, TIP3P-FB, and TIP4P-FB water models. (A) and (B) show the profile for the first chain (sn-1), while the (C) and (D) shows the second chain (sn-2). Experimental values are reproduced from Seelig & Seelig (1974), Seelig & Waespe-Sarcevic (1978), Douliez, Léonard & Dufourc (1995), Li et al. (2017). In most cases, the values from the simulations are so similar that the points lie on top of each other. The numbering of the positions on the acyl chains is illustrated in Fig. 2.

The calculated acyl C–H order parameters, and those determined experimentally, are plotted in Fig. 4. The acyl C–H order parameters are generally insensitive to the water model and simulations using any of the three models predict order parameters that are in good agreement with the experimental values. This trend holds for the acyl groups in the upper region of the chain that are close to the water layer, which indicates that the lipid–water interface is similar for all three water models.

**Figure 5:**
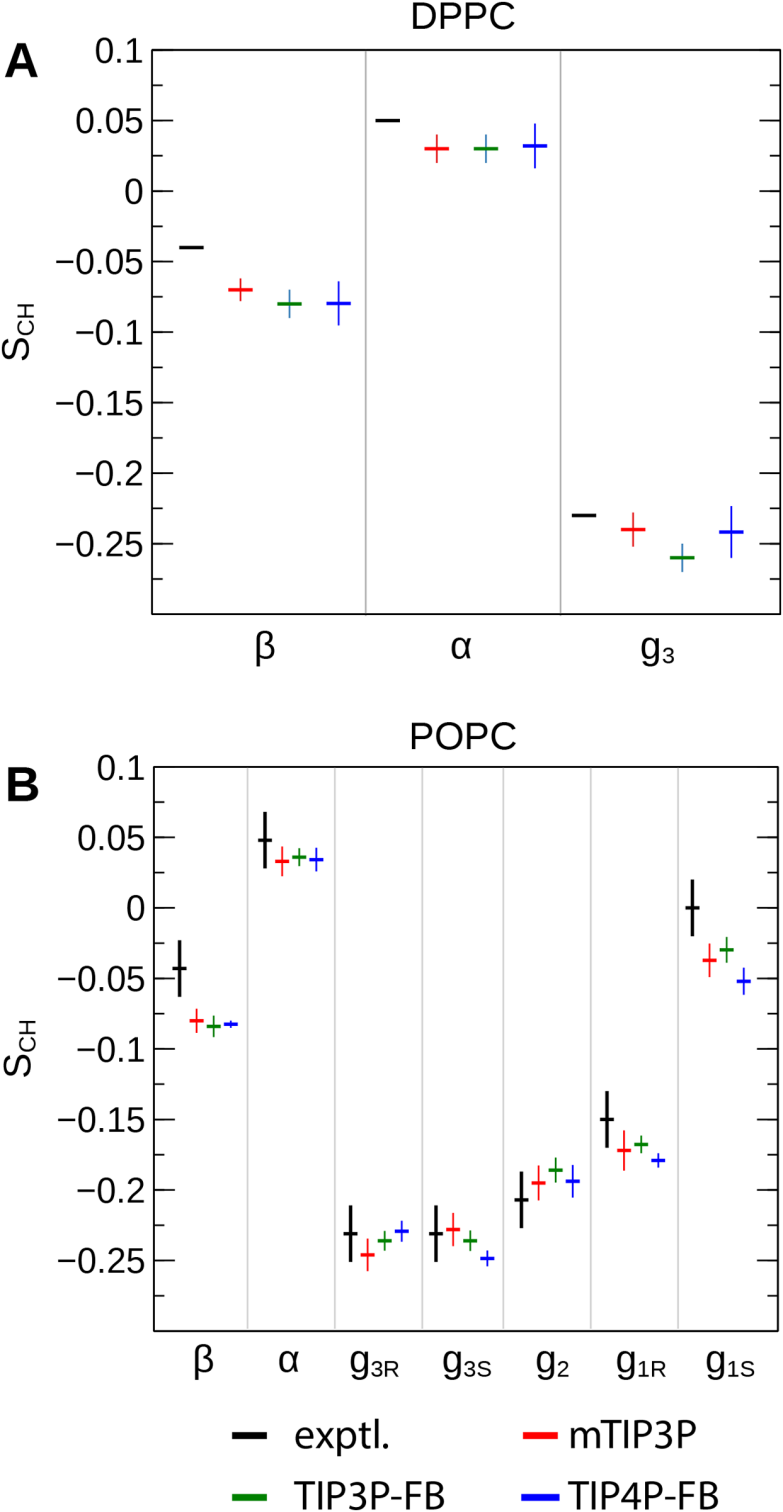
NMR order parameters (*S_CH_*) for the lipid headgroups of the (A) DPPC and (B) POPC bilayers calculated from simulations of the bilayers with the mTIP3P, TIP3P-FB, and TIP4P-FB water models. Experimental values for DPPC are taken from Gally, Niederberger & Seelig (1975). Experimental values for POPC are taken from Ferreira et al. (2013). The assignments of the order parameters follow those presented in Botan et al. (2015). The labeling of the positions of the headgroup is illustrated in Fig. 2.

The order parameters of the atoms in the lipid headgroups are presented in Fig. 5. These positions tend to show a lower degree of order than the acyl chains (i.e., |*S*_CH_| < 0.1). All models are in reasonably good agreement with the experimental values. The results of the TIP3P and TIP3P-FB model simulations are in close agreement, while the TIP4P-FB model predicts incrementally more negative order parameters for the POPC glycerol positions (i.e., *g*_1_, *g*_2_, and *g*_3_).

### Bilayer electron density and scattering

**Figure 6:**
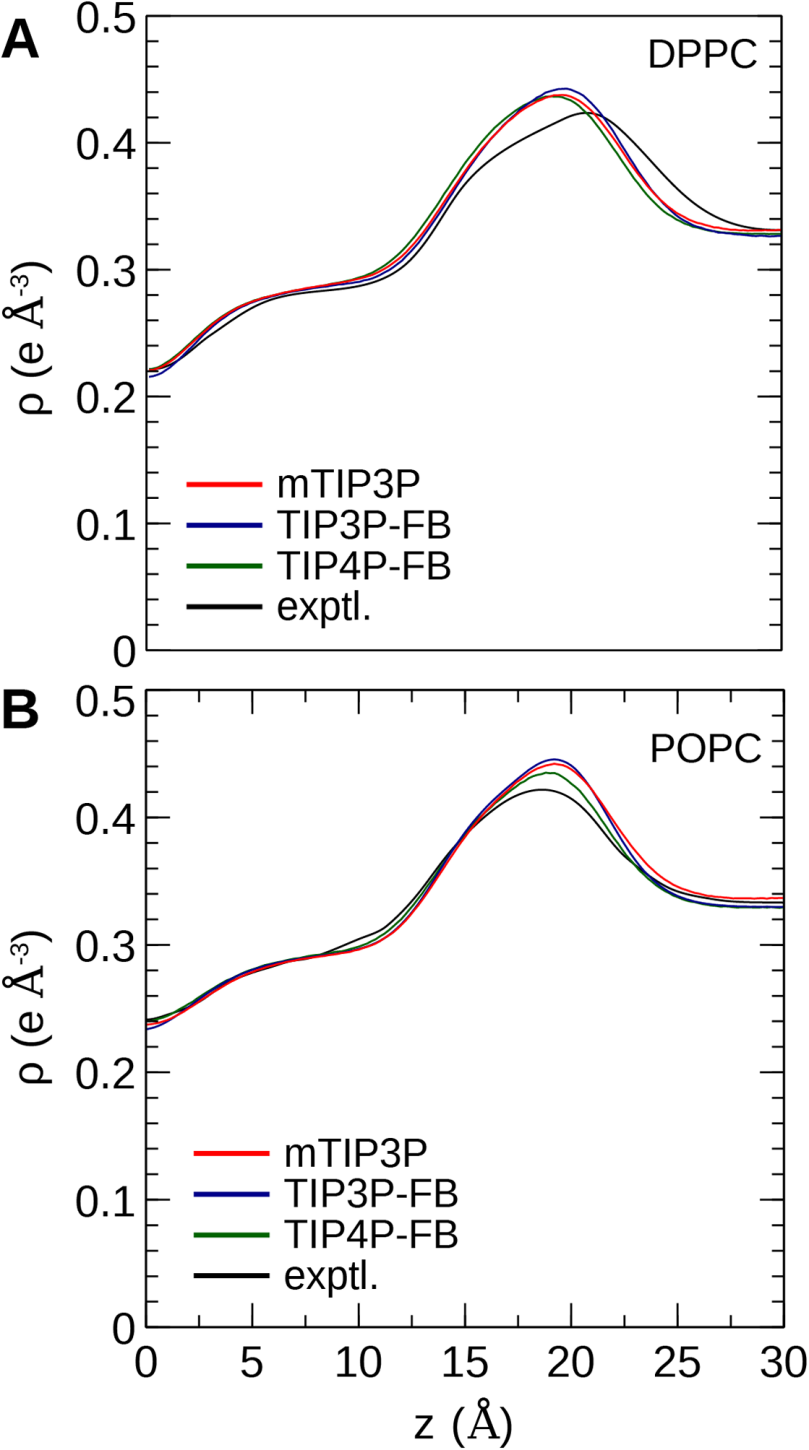
Electron density profile for (A) DPPC and (B) POPC bilayers calculated from simulations using the CHARMM36 lipid force field and the three water models. The experimental curves are reproduced from Kučerka, Tristram-Nagle & Nagle (2006a, 2006b).

The calculated electron density profiles for the three water models are presented in Fig. 6. The profiles are similar for all three water models, although, for the DPPC bilayer, the electron density maximum is slightly higher and occurs at a higher value of *Z* for the TIP3P-FB model in comparison to the mTIP3P model. This indicates that there is an incremental thickening of the bilayer (1 Å) when this water model is used. The experimental electron density profile for the POPC bilayer shows that the bilayer is incrementally thinner and has a lower maximum than the simulated profiles.

**Figure 7:**
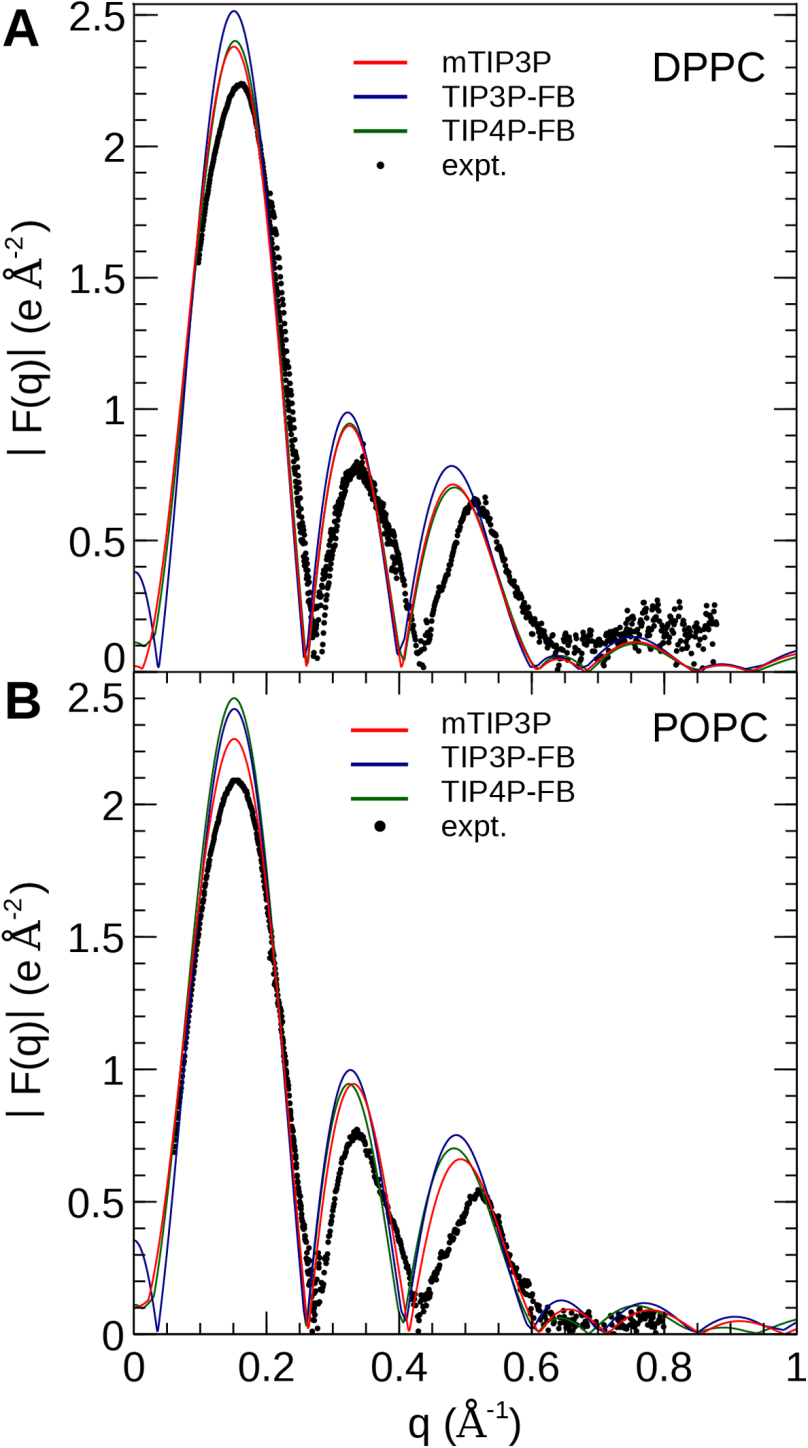
X-ray scattering profiles of (A) DPPC and (B) POPC lipid bilayers calculated from the simulated electron density profiles. The experimental profile is reproduced from Kučerka et al. (2008).

The electron density profiles of the two bilayers were transformed into reciprocal space (*F*(*q*)) so that X-ray scattering curves could be compared directly to the experimental profiles determined from oriented multilayers and unilamellar vesicles (Kučerka et al., 2008). The amplitudes of the scattering form factors (|*F*(*q*)|) were calculated from the electron density profiles using the relation,

(7) $$|F(q)| = \left| {\int_{-L/2}^{L/2} \left[ {{\rm{\rho }}(z)-{{\rm{\rho }}_w}} \right]\left({\cos (qz) + i\sin (qz)} \right){\rm{d}}z} \right|$$

where ρ_*w*_ is the electron density of the bulk solvent (i.e., water) and *q* is the *z*-component of the scattering vector. These curves are presented in Fig. 7. The curves calculated using all three water models show only subtle differences, consistent with the similar electron density profiles. In comparison to the experimental scattering curves, the positions of the nodes are shifted to incrementally smaller values of *q* for all three water models and both lipids, but otherwise, all three models are consistent with the X-ray scattering data.

**Figure 8:**
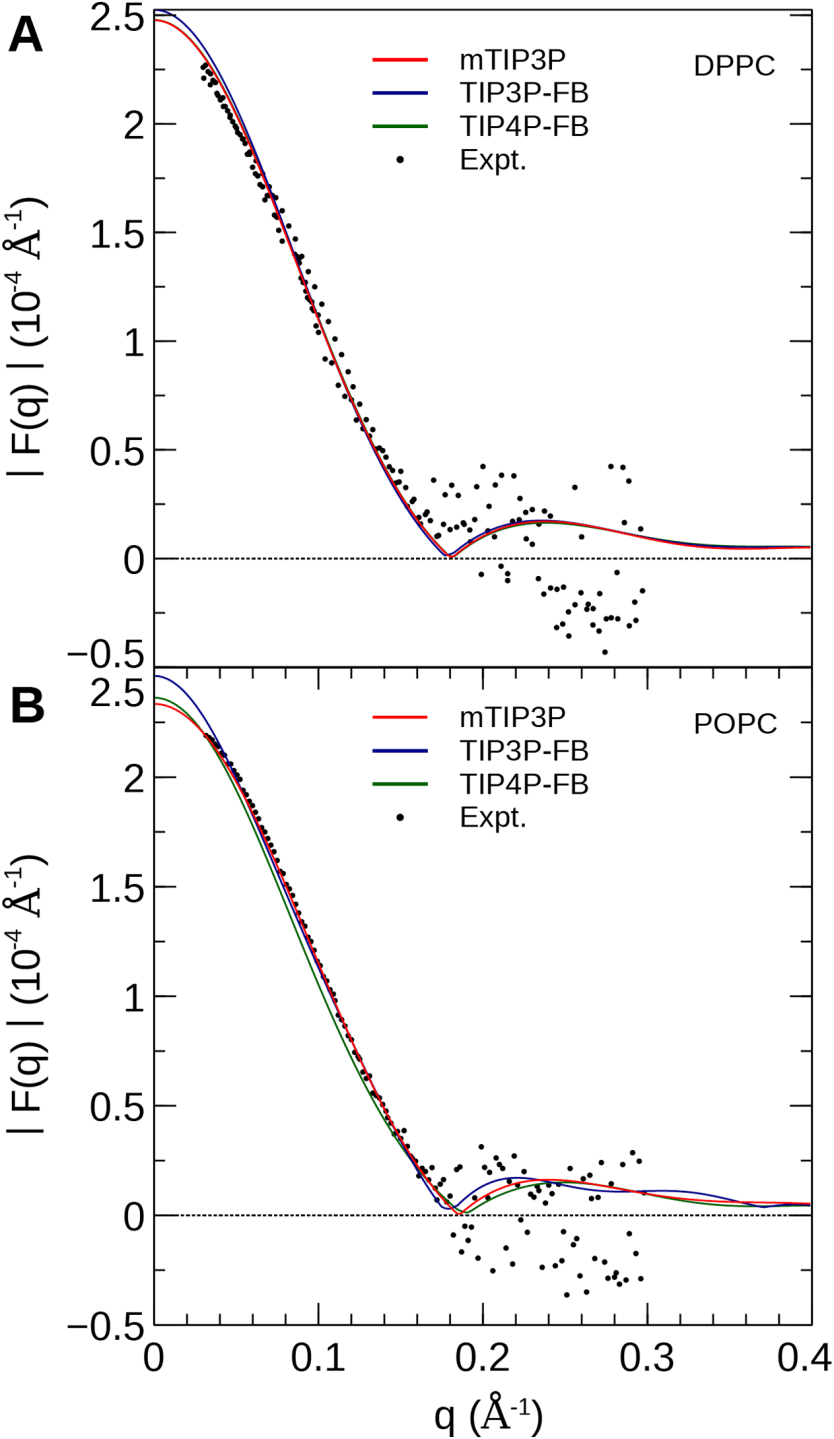
Neutron scattering profiles of (A) DPPC and (B) POPC lipid bilayers calculated from the simulated neutron scattering length profiles. The experimental profile is reproduced from Kučerka et al. (2008).

The NSLD profile of a membrane is dominated by the sharp difference in the NSL of protons in the lipids (*a_H_* = −3.74 fm) relative to the deuterons of the heavy water solvent (*a_D_* = 6.67 fm). As a result, the neutron scattering curve is sensitive to the thickness of the hydrocarbon layer of the bilayer and the depth of penetration of water into the bilayer (Kučerka et al., 2008). The reciprocal-space neutron scattering curves were calculated from the NSL density profiles calculated from the simulations (Kučerka, Katsaras & Nagle, 2010). These curves are presented in Fig. 8. For both lipid types, the neutron scattering curves calculated using all three models are in good agreement with the experimental scattering curves in the interval where reliable experimental data is available (0.2 Å^−1^ < *q* < 0.2 Å^−1^). This is consistent with the density profiles, which show only small variations in the bilayer thickness when the water model is changed.

### Membrane dipole potential

**Figure 9:**
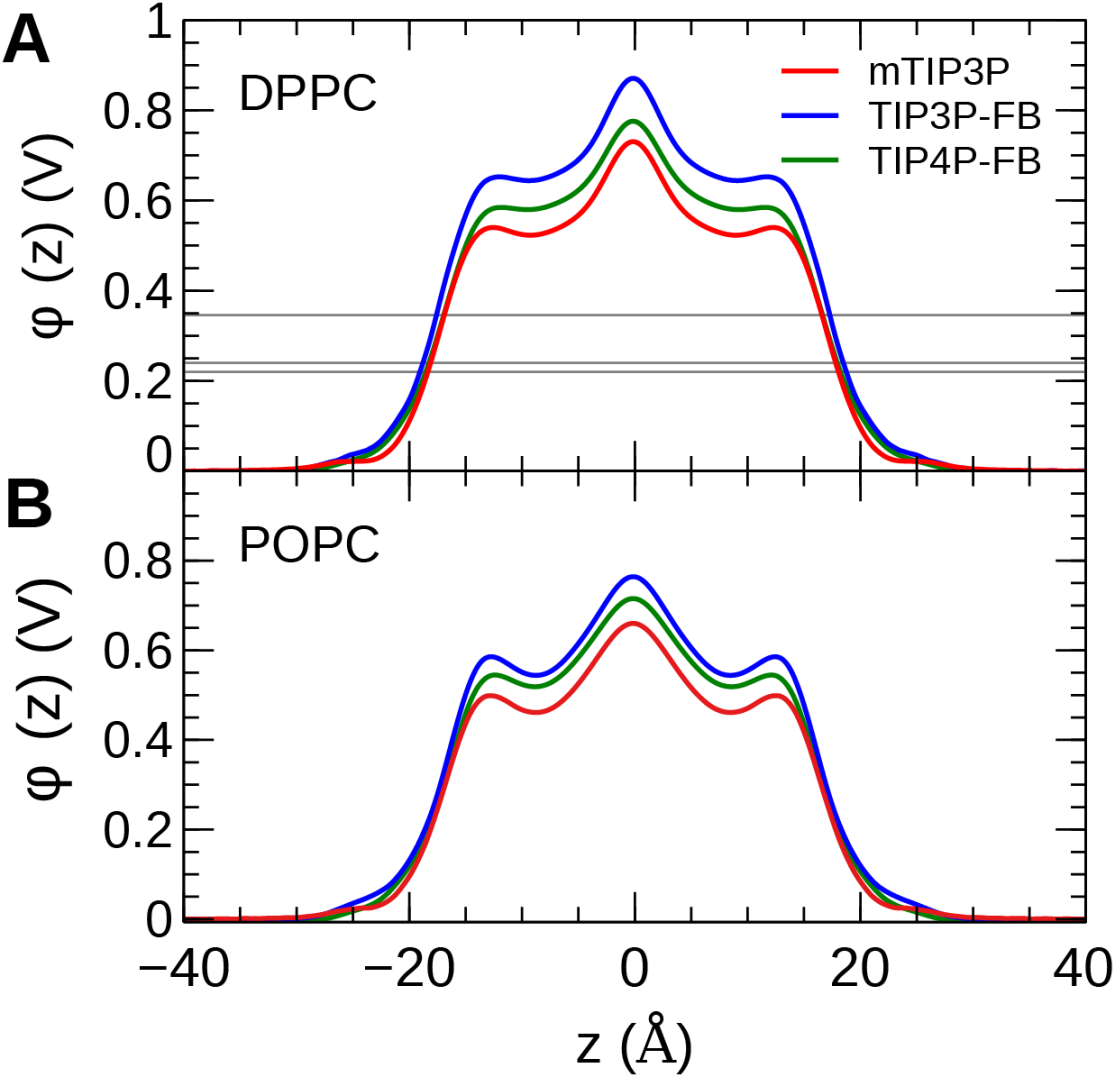
The membrane dipole potential (ϕ) calculated for the three water models of (A) DPPC and (B) POPC lipid bilayers. The experimental values for DPPC from Gawrisch et al. (1992), Schamberger & Clarke (2002), and Peterson et al. (2002) are indicated by the gray horizontal lines.

The calculated MDP profiles are presented in Fig. 9. The CHARMM36/mTIP3P force field overestimates the MDP; for DPPC, the maximum of the MDP is 730 mV, while the experimental estimates range from 220 to 346 mV. High MDPs are also predicted for the CHARMM36/mTIP3P force field POPC force field. The use of the TIP3P-FB and TIP4P-FB models result in a systematic increase in the MDP for both the DPPC and POPC bilayers, where the maximum for the MDP is roughly 100 and 50 mV higher, respectively. Most of this difference originates from changes in the electrostatic potential at the lipid–water interface, which is greater when the more polar FB models are used.

### Water permeability

**Figure 10:**
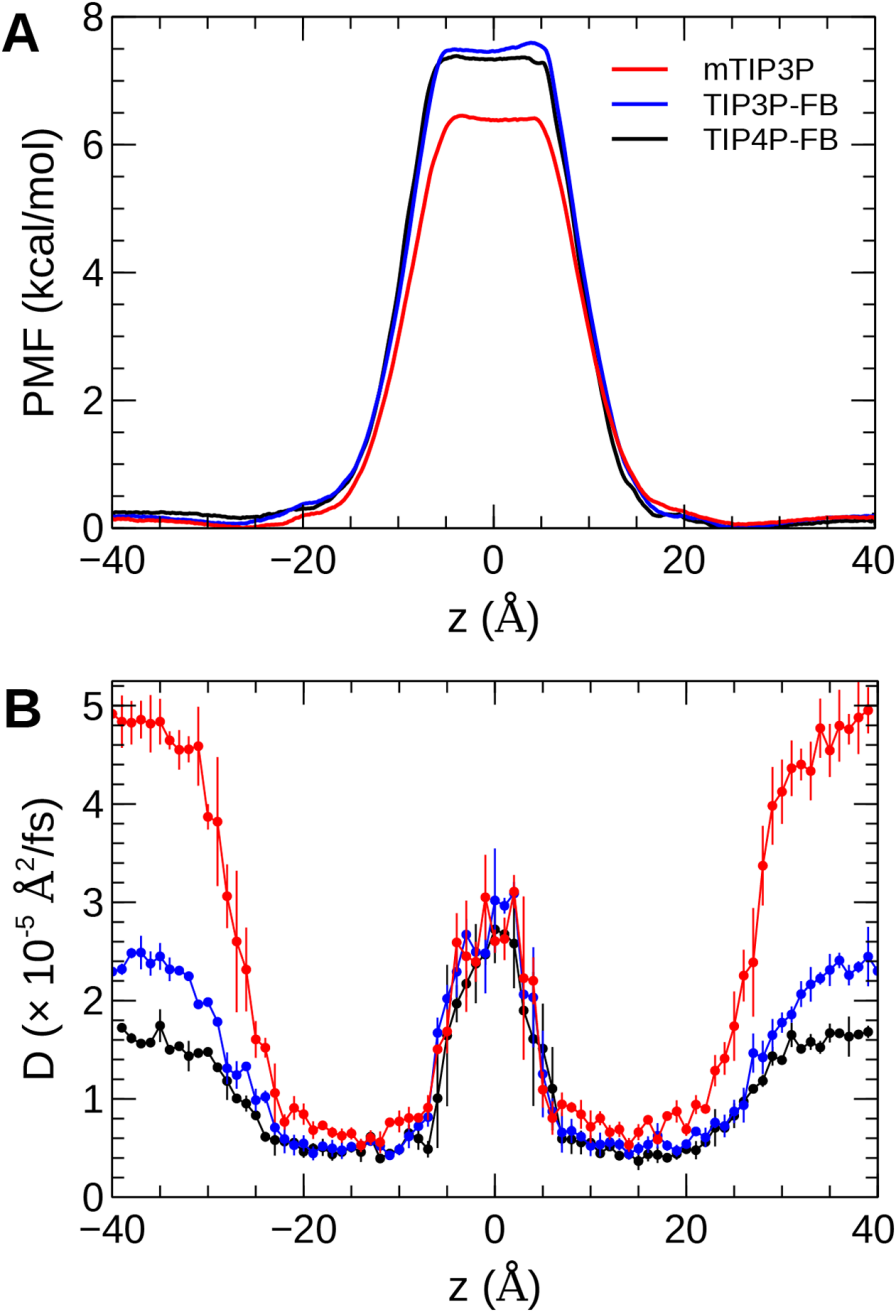
The (A) PMF and (B) diffusivity profiles for a water molecule permeating a pure POPC bilayer at 298 K.

The calculated PMF’s and diffusivity profiles of water molecules permeating through the bilayer are presented in Fig. 10. The water permeabilities of the POPC bilayer calculated using these profiles are presented in Table 4.

**Table 4:** Water permeability of a pure POPC bilayer using the CHARMM36 lipid force field and the three selected water models.

Model	*P_m_* (× 10^−3^ cm/s)
mTIP3P	4.37 ± 0.03
TIP3P-FB	0.76 ± 0.02
TIP4P-FB	0.88 ± 0.01
Expt.	13.0 ± 0.44

**Note:**

The experimental value is taken from Mathai et al. (2008).

The effect of the water model on the diffusivity is apparent in transmembrane diffusivity profile (|*z*| > 20 Å). The TIP3P water model has a viscosity coefficient that is much lower than the experimental value (η_TIP3P_ = 0.321 mPa·s vs η_expt_ = 0.896 mPa·s), so its rate of diffusion in the solution and at the lipid–water interface is unrealistically fast (${D_{\rm{H_2}O}} = {\rm{}}6.05{\ \rm{c}}{{\rm{m}}^2}/s$). These results are in line with previous simulations (Issack & Peslherbe, 2015; Awoonor-Williams & Rowley, 2016). The TIP3P-FB and TIP4P-FB water models have viscosity/self-diffusion coefficients that are much closer to the experimental values, and the diffusivity of the permeating water molecule is lower accordingly. Using these more realistic water models, the water solute diffuses at a faster rate at the center of the bilayer than in solution, opposite to the trend predicted using the mTIP3P model.

The PMF when the permeating water molecule is at the center of the bilayer is 6.4 kcal/mol when the mTIP3P model is used but is approximately 7.4 kcal/mol for the TIP3P-FB and TIP4P-FB models. This can be connected to the Gibbs energy of transfer of a water molecule between liquid water and liquid hexadecane. The model for hexadecane uses the same non-bonded parameters as the aliphatic sections of the lipid chains, making it an appropriate model for the partitioning of a water molecule between the aqueous phase and the center of the bilayer. The results from the FEP/TI calculation of the Gibbs energy of transfer are presented in Table 5. The electrostatic component of the Gibbs energy of transfer is about 1 kcal/mol larger for the TIP3P-FB and TIP4P-FB water models. This larger thermodynamic penalty for transferring a water molecule into hexadecane is consistent with the FB models having higher PMF’s of permeation than for the TIP3P model. This ultimately reflects that the TIP3P water model has a smaller excess chemical potential than the TIP3P-FB and TIP4P-FB models (Table 6), resulting in a smaller thermodynamic penalty to remove a TIP3P-model water molecule from the aqueous phase. The experimental estimates of ${\rm \Delta{\rm{\mu }}_{{{\rm{H}}_2}{\rm{O}}}}$ are consistently lower than those predicted by the TIP3P-FB and TIP4P-FB models. It should be noted that the simulations used in this study use force-based switching on the Lennard-Jones potential, while the OpenMM code used to parameterize the FB models use a potential-based switching function. Complete tables of the calculation of the transfer energies are included in Tables A1 and A2.

**Table 5:** Gibbs energy of transfer of one water molecule from liquid water to liquid hexadecane.

Model	mTIP3P	TIP3P-FB	TIP4P-FB	Expt.
Elec.	8.19 ± 0.06	9.54 ± 0.04	9.45 ± 0.08	–
Disp.	−0.68 ± 0.06	−0.89 ± 0.09	−0.82 ± 0.14	–
Rep.	0.00 ± 0.38	−0.07 ± 0.50	− 0.17 ± 0.41	–
Total	7.51 ± 0.50	8.58 ± 0.63	8.46 ± 0.63	5.98

**Note:**

All values are in units of kcal/mol. The experimental value is taken from Abraham et al. (1990).

**Table 6:** Calculated and experimental excess chemical potentials of water.

Model	Δμ (kcal/mol)
mTIP3P	−6.33 ± 0.07
TIP3P-FB	−7.46 ± 0.05
TIP4P-FB	−7.50 ± 0.07
Expt.	−6.32, Ben-Naim & Marcus (1984) −5.74 Hermans, Pathiaseril & Anderson (1988)

## Discussion

The calculated headgroup areas and compressibilities showed limited variation with the water model. Experimental values of the headgroup area are typically derived from a combination of estimates of bilayer properties, such as bilayer thickness, volume per lipid, etc. As a result, a wide range of values has been reported for DPPC and POPC lipids (Poger, Caron & Mark, 2016). Although the uncertainty in the experimental values makes it difficult to conclude that one water model yields improved headgroup areas and compressibilities, we can conclude that these properties are similar for all three water models and are within the margin of uncertainty of widely-used experimental values.

The calculations of the X-ray and neutron scattering profiles generally indicate that all three water models yield structural distributions that are generally consistent with the experimental form factors, although the nodes of the X-ray scattering profile of DPPC bilayer are at systematically smaller values of *q* for all three water models. This suggests that the lipid model would have to be adjusted in order to improve agreement between the simulated profiles and the computationally-predicted profiles because there are only subtle differences in the scattering profiles calculated using the three water models.

Botan et al. (2015) had previously shown that the CHARMM36/mTIP3P model was among the most effective force fields for predicting lipid headgroup order parameters. The lipid order parameters calculated from the simulations presented here were generally insensitive to the water model and were all in reasonable agreement with the experimental values determined using NMR. These data provide some of the more direct measures of the structure of the bilayer, especially at the lipid–water interface, so the success of the TIP3P-FB and TIP4P-FB models in calculating order parameters is particularly encouraging. The order parameters of the acyl positions are essentially the same for all three water models.

The MDP was overestimated by all models. Simulations with the FB water models overestimate the MDP to an even greater degree than the mTIP3P model. This suggests that the description of the MDP calculated using the CHARMM36 lipid force field cannot be significantly improved by using improved water models. The CHARMM-Drude polarizable force field for lipids predicts more moderate values for the MDP (≈ 0.56 V at the center of the bilayer) (Li et al., 2017). Harder and Roux attributed the improved performance of these polarizable force fields to the polarization of the upper portions of the acyl chains by the water–headgroup interface, which attenuates the increase in the profile in the lipid-headgroup region (Harder, MacKerell & Roux, 2009). This induced polarization effect is not captured by non-polarizable models like CHARMM36; however, the description of other physical properties of the bilayer does not appear to be negatively affected by the neglect of this effect.

The water permeability of a lipid bilayer is notably sensitive to the water model. The rate of permeation of water across a pure POPC bilayer has been measured at 13 × 10^−3^ cm/s (Mathai et al., 2008), indicating that water molecules are able to cross a membrane at a slow but significant rate. The permeability calculated using the mTIP3P model underestimates this rate by a factor of 3, while the FB models underestimate the rate by a factor of 15–17.

The factor that affects the rate of permeation most significantly is the height of the barrier in the PMF, which is significantly higher for the FB models than for mTIP3P. All models significantly underestimate the experimental solubility of water in hexadecane, although the FB models overestimate the water–hexadecane transfer energy to a larger degree than the mTIP3P model. These appear to be rooted in the spuriously large excess chemical potentials for the FB models, which we calculated to be −7.46 and −7.50 kcal/mol. In comparison, Shirts & Pande (2005) showed that the chemical potentials of SPC, SPC/E, TIP3P, TIP4P, and TIP4P-Ew ranged from −6.10 to −7.05 kcal/mol, which are closer to the experimental estimates of −5.7 and −6.3 kcal/mol. Limitations of the force field combination rule and neglect of induced polarization have also been proposed to cause the solubility of water in alkane solvents to be underestimated (Ballal et al., 2014; McDaniel & Yethiraj, 2016). Reparameterization of the alkane–water non-bonded parameters may be needed to capture the correct water permeability of the bilayer using these models. Because this issue stems specifically from the high excess chemical potential of water in the ForceBalance models, it is unlikely to affect the permeability of other non-ionic solutes, although solutes that permeate in complex with water molecules may experience a higher barrier.

Piggot, Piñeiro & Khalid (2012) observed that simulations of DPPC lipid bilayers in GROMACS using the TIP3P water model and a potential-based switching function resulted in a transition to an ordered phase. This issue did not appear in our simulations, which used force-based switching functions in NAMD. Despite our success, Piggot, Piñeiro & Khalid (2012) results suggest that the TIP3P-FB and TIP4P-FB should still be used cautiously with the CHARMM36 lipid model because subtle differences in simulation options can result in significant differences in bilayer properties, so our results may not apply when different non-bonded cutoffs, switching options, etc. are used.

## Conclusions

The DPPC and POPC lipid bilayers were simulated using MD using the TIP3P-FB and TIP4P-FB water models and compared to the results from simulations using the mTIP3P water model. The headgroup area, compressibility, X-ray and neutron scattering profiles, and acyl-chain deuterium order parameters were compared to experimental values. All three models yielded similar results, suggesting that the CHARMM36 model can be used with any of these water models without modification for the simulation of the structure and dynamics of lipid bilayers. This could be advantageous in some instances, as the dielectric constant and viscosity of water simulations using the TIP3P-FB and TIP4P-FB models are closer to the experimental values than when the mTIP3P model is used. The temperature dependent properties of the TIP4P-FB model water are also improved, which could be an advantage in the simulation of temperature dependent properties of water–lipid systems.

More significant differences were apparent in the water permeability of the bilayers. The PMF and diffusivity of a permeating water molecule were calculated along the transmembrane axes and these data were used to calculate the permeability of the bilayer using the solubility–diffusion model. Although this model is approximate, the TIP3P model was in closer agreement with the experimental estimates than the TIP3P-FB and TIP4P-FB models. This difference stems from the higher PMF when the permeating water molecule is in the middle of the bilayer when the TIP3P-FB and TIP4P-FB water models were used. In turn, this barrier reflects a spuriously low water–alkane partition coefficient for the TIP3P-FB and TIP4P-FB models due to their high excess chemical potentials. This could, in principle, be improved by modifying the water–acyl non-bonded parameters for these models, but this issue is unlikely to be significant for simulations of most of the structural and dynamic properties of lipid bilayers or the permeation of other solutes.

## Appendix

**Table A1:** Calculated excess chemical potential of water.

Model	Elec.	Disp.	Rep.	Total
mTIP3P	−8.41 ± 0.02	−2.72 ± 0.00	4.80 ± 0.05	−6.33 ± 0.07
TIP3P-FB	−9.78 ± 0.02	−2.44 ± 0.00	4.76 ± 0.02	−7.46 ± 0.05
TIP4P-FB	−9.68 ± 0.05	−2.77 ± 0.00	4.95 ± 0.02	−7.50 ± 0.07
Expt.			−6.32^a^, −5.74^b^	

**Notes:**

Values are in kcal/mol.

a Ben-Naim & Marcus (1984).

b Hermans, Pathiaseril & Anderson (1988).

**Table A2:** Absolute solvation energy of a water molecule in liquid hexadecane.

Model	Elec.	Disp.	Rep.	Total
mTIP3P	−0.22 ± 0.04	−3.40 ± 0.06	4.80 ± 0.43	1.18 ± 0.53
TIP3P-FB	−0.24 ± 0.02	−3.33 ± 0.09	4.69 ± 0.52	1.12 ± 0.63
TIP4P-FB	−0.23 ± 0.03	−3.59 ± 0.14	4.78 ± 0.43	0.96 ± 0.60

**Note:**

Values are in kcal/mol.

## Supplemental Information

10.7717/peerj.5472/supp-1 Supplemental Information 1 Data for plots of neutron scattering profiles.

10.7717/peerj.5472/supp-2 Supplemental Information 2 Data for plots of X-ray scattering profiles.

10.7717/peerj.5472/supp-3 Supplemental Information 3 Sample input, topology, and input files for NAMD simulations.

## Additional Information and Declarations

### Data Availability

The following information was supplied regarding data availability: The raw data are provided in the Supplemental Files.
